# Interaction Between Concrete and FRP Laminate in Structural Members Composed of Reused Wind Turbine Blades Filled with Concrete

**DOI:** 10.3390/ma17246186

**Published:** 2024-12-18

**Authors:** Anna Halicka, Lidia Buda-Ożóg, Mirosław Broniewicz, Łukasz Jabłoński, Joanna Zięba, Filip Broniewicz

**Affiliations:** 1 Department of Building Structures, Faculty of Civil Engineering and Architecture, Lublin University of Technology, ul. Nadbystrzycka 38d, 20-618 Lublin, Poland; l.jablonski@pollub.pl; 2 Department of Building Structures, Faculty of Civil Engineering and Environmental Engineering, Rzeszow University of Technology, Poznańska 2, 35-084 Rzeszów, Poland; lida@prz.edu.pl (L.B.-O.); j.zieba@prz.edu.pl (J.Z.); 3 Department of Building Structures and Structural Mechanics, Faculty of Civil Engineering and Environmental Sciences, Bialystok University of Technology, Wiejska 45A, 15-351 Bialystok, Poland; m.broniewicz@pb.edu.pl (M.B.); filip.broniewicz@pb.edu.pl (F.B.)

**Keywords:** wind turbine blade, recycling, FRP laminate, hybrid structural element, concrete, roughness, push-off test, bond strength

## Abstract

The lifecycle of wind turbine blades is around 20–25 years. This makes studies on the reuse of dismantled blades an urgent need for our generation; however, their recycling is very difficult due to the specific makeup of their composite material. In this study, the authors determined a concept for the reuse of turbine blade sections filled with concrete for geotechnical structures, retaining the walls, piles, or parts of their foundations. Working out detailed structural solutions to the above problem should be preceded by the identification of material parameters. In particular, getting to know the interface stress-strain characteristics is crucial. Therefore, this research focuses on the cooperation between recycled FRP composites and concrete in load-carrying, including experiments and numerical analyses. Regarding the two types of destructive stress, which may occur at the interface under both compression and bending, two types of tests were executed: the ‘push-out test’, modelling the interface’s answer to shear stress, and the ‘pull-off test’, demonstrating the interface’s reaction to normal stress. Additionally, the strength parameters of the materials used were tested. The numerical model for the push-out process was calibrated on the basis of the tests, and this way the shear bond strength and the coefficient of friction between the concrete and the recycled FRP laminate were assessed.

## 1. Introduction

The need to reduce reliance on conventional fuels and replace them with renewable energy is currently an urgent issue. This situation is forced by the principle of sustainable development, which consists of meeting the present generation’s needs without impacting those of future generations. Therefore, activities within the framework of sustainable development focus on the responsible management of natural resources and the protection of the environment. Wind turbines constitute an increasingly important source of renewable energy. They provide a clean energy source, emitting no greenhouse gases and no waste products during operation. The most popular are three-bladed horizontal-axis wind turbines (HAWT). The blades range in length from 20 to 80 m, while the hub height of horizontal-axis wind turbines is about 90 m.

Materials commonly used for wind turbine blades are composites of glass or carbon fibers, as well as hybrid ones in a thermoset polymer matrix (mainly epoxy). More often, for wind turbine blades, a glass/epoxy composite is used, which typically contains up to 75% glass in terms of weight [1]. The less-often-used carbon fiber composite has a higher tensile strength and stiffness and lower density than the glass fiber one. The fibers are used for reinforcement in composites, being responsible for the stiffness and tensile and compressive strength of the structural material. The composite material is characterized by a high strength-to-weight ratio, high flexural, impact, and fatigue strengths, resistance to corrosion, and high damping property.

One disadvantage concerning wind turbine is that the average lifecycle of a wind turbine is approximately 20–25 years. Furthermore, older turbine models demand substitution with more efficient ones. Delaney et al. [2] presented a scenario forecasting that the total number of wind turbine blades demanding replacement up until 2025 will exceed one million. Their deposition requires increasingly more space and is becoming increasingly expensive. These facts make studies on the reuse of dismantled wind turbine parts an urgent need for our generation. Such studies should focus on developing a circular economy model for turbine blade use.

The blades are difficult to recycle because the fibers cannot be simply extracted and used in new materials [3,4]. This is why efforts have focused on the reuse of crushed, shredded, or milled wind turbines as a component for new material [3,4].

Another different approach is based on the concept that the turbine blades may not only be a source of components for new materials but also a source of structural elements (upcycling). Small pieces, in the order of tens of centimeters, can serve as boards, for example, in furniture production [5,6] or as floorboards. Bigger elements, including whole-blade cross-sections (or parts thereof), have been used to create public relaxation infrastructure, e.g., benches [7], playground equipment, and climbing walls [6]. It is also possible to make engineering components, such as culverts and manholes, barriers and fencing, posts and bollards [8], as well as farm infrastructure features (planters, animal pen fences, grain partition walls, feed bunks, etc.) [8,9].

The challenge of creating more important structures than those listed above has also been accepted. A concept house with its walls and roof made from wind turbine blades has been developed [8,10,11]. A three-phase electrical transmission tower has also been designed and tested in detail [12,13,14,15]. Bus shelters and noise barriers have also been constructed [8,9,16]. Footbridges, in which wind turbine blades constitute bearing members, have been designed and constructed [17,18,19,20,21]. Marine use is also possible. Closed, hollow blades (placed vertically or horizontally) can be used as floating buoys, as well as the components of piers, docks, or platforms carrying photovoltaic solar arrays [9].

**Figure 1:**
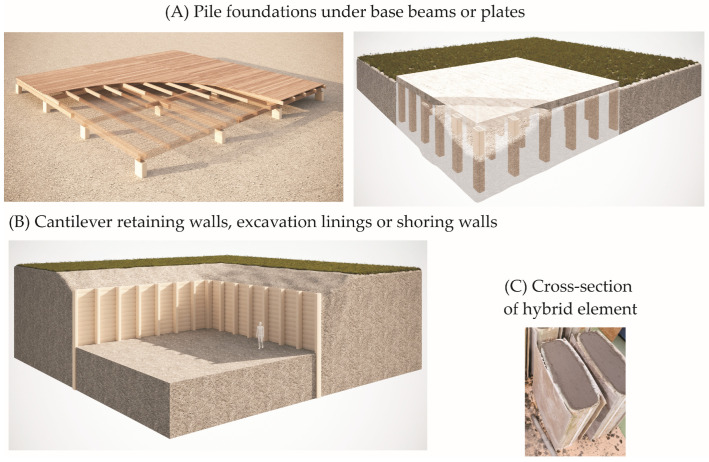
Concepts of reuse of turbine blade sections filled with concrete: (**A**) pile foundations under base beams or plates (piles made of Vestas-type spar caps filled with concrete), (**B**) cantilevered retaining walls, excavation linings, or shoring walls (cantilever beams made of Vestas-type spar caps filled with concrete and plating made of fragments of LM blades), (**C**) cross-section of hybrid element.

The authors determined the conception for creating hybrid structural elements made of turbine blade sections filled with concrete, dedicated to geotechnical structures such as retaining walls, piles, or parts of foundations [22,23] (Figure 1). The sections of turbine blades may serve in such solutions as the formwork and protect the concrete and steel reinforcement from the physical and chemical influence of the surrounding environment.

Similar hybrid structural elements made of steel tubes filled with concrete are commonly used in civil engineering. Furthermore, hybrid elements made with new (not recycled) circular [24,25,26,27,28,29,30] or elliptical [31,32] FRP tubes have been created and tested. The corrosion resistance and high ductility required in seismic regions were identified as assets in the abovementioned papers. What is more, the FRP tube laterally confines the concrete strain, increasing the compressive and flexural bearing capacity [26].

The advantages of hybrid columns would be greater if the concrete and FRP tube cooperated in terms of load-bearing. This is only possible to achieve if there is strong enough adhesion at the interface between the concrete and the FRP tube. Therefore, identification of the interface stress-strain characteristics is crucial. However, this problem has been studied mostly in relation to the bond between the concrete and FRP built with the use of adhesives (while strengthening the beams or plates with FRP strips or sheets as well as the concrete columns wrapped with FRP sheets, e.g., [33,34,35,36,37]). The subject of direct adhesion between FRP laminate and concrete (without adhesive) was studied in relation to the bond between concrete and FRP bars used as internal reinforcement, e.g., [38,39,40]. The problem is also crucial for hybrid columns, particularly in the end sections, transferring the load onto the column. If the concrete slips relative to the FRP tube, this may lead to separation of the originally connected elements, possibly overloading the tube. The problem—regarding new (not recycled) FRP tubes of 100–165 mm in diameter—was studied for different types of concrete and reported in the papers [41,42,43,44,45], where the ‘load-slip’ curves, bond strength, and coefficient of friction were identified under static load [41,42,44,45] (including in elevated temperatures [41,45]) and cyclic load [43].

The detailed outline of the structures presented in Figure 1 demands the examination of the behavior of recycled FRP tubes filled with concrete under different loads. Our previous paper [23] presented the performance of a hybrid element under flexure. The present paper describes the study outlining the parameters controlling the bond between turbine blade elements and concrete (the shear bond strength in the interface between the concrete and recycled FRP tube and the coefficient of friction). Such data are necessary for the modeling and structural design of hybrid structures, which will be the next step in our study. This should be emphasized that the study was inspired by a company dealing with the storage and recycling of wind turbine wastes. The general aim was to prove that the proposed structures may serve as good classical civil engineering structures.

In summary, the novelty of the presented research lies in the experiments to reuse large sections of wind turbine blades filled with concrete for geotechnical purposes. The cooperation between reused composite and concrete, which is necessary in hybrid structures, was tested. The cooperation parameters (the adhesion and coefficient of friction values) were determined on the basis of tests and numerical analyses.

## 2. Materials and Methods

The program of research covered tests focused on investigating the physicomechanical phenomena occurring at the interface between FRP laminate acquired from recycled turbine blades and concrete as the result of their cooperation in a test structure.

**Figure 2:**
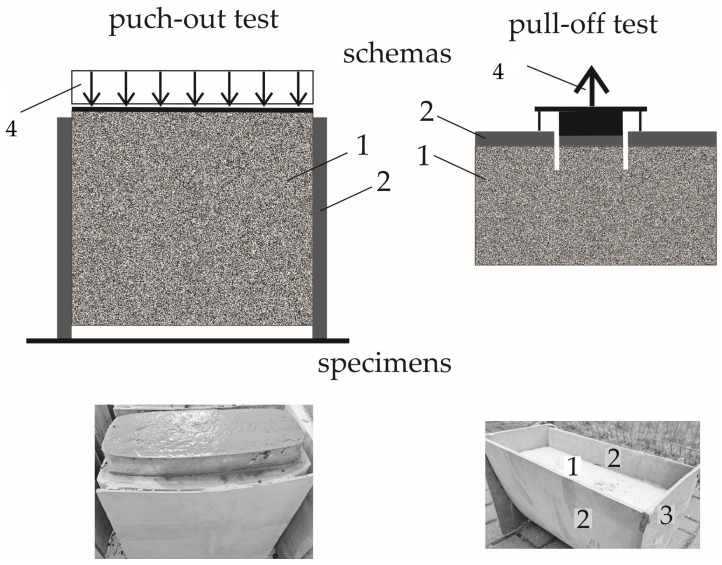
Schemas of bond tests and specimens prepared for testing: 1—concrete, 2—composite part of turbine blade, 3—closing board enabling the concreting, 4—applied load.

Regarding the fact that two types of destructive stress may occur at the interface due to the loading of the structure, two types of tests were executed (Figure 2): a ‘push-out test’, modeling the interface’s response to applied shear stress (shear bond strength), and a ‘pull-off test’, showing the interface’s reaction to normal stress (tensile bond strength).

Additionally, the strength parameters of the materials used (FRP laminate acquired from turbine blades and concrete) were tested.

The research program was complemented with numerical simulations of the conducted push-out test.

### 2.1. Specimen Preparation

**Figure 3:**
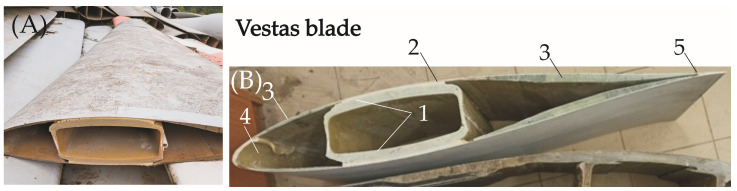
Dismantled turbine blade parts used in the experiment: (**A**) turbine blades in the scrap heap; (**B**) section cutout of turbine blade for testing: 1—spar cap, 2—adhesive, 3—aerodynamical shell (outer balsa, polyurethane foam filling, and inner FRP composite), 4—composite strengthening of leading edge, 5—run-off edge.

Parts of Vestas turbine blades, namely fragments of spar caps (inner FRP laminate elements of the box section), were used as recycled FRP tubes, being the formworks for the concrete (Figure 3). The spar caps are the main load-bearing elements of turbine blades, withstanding the wind action. They are surrounded by the outer sandwich shells profiled according to aerodynamic rules.

**Figure 4:**
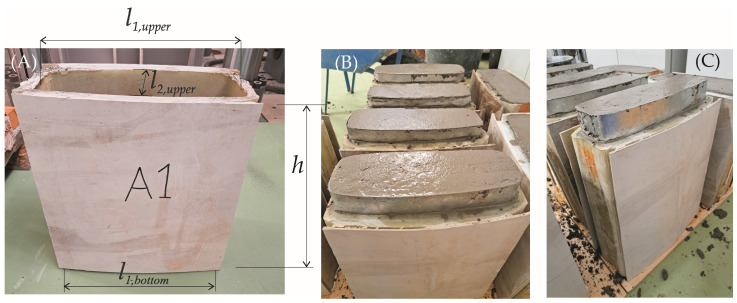
Specimens prepared for push-out testing: (**A**) FRP laminate tube with marked main internal dimensions for push-off testing, (**B**,**C**) tubes filled with concrete.

First, the turbine blade was cut into sections similar to those shown in Figure 3B. Then, the parts of the aerodynamic shells not glued to the spar caps were cut off. The obtained four FRP laminates of the box-shaped elements (FRP tubes) were used in the push-out testing (Figure 2 and Figure 4), while the remaining parts of the aerodynamic shell were used for the pull-off testing after placing concrete into the space between the aerodynamic shell and the closing decks (Figure 2).

The internal dimensions of the four tested FRP tubes are shown in Table 1. The specimens for push-out testing were prepared by filling them with concrete. During concreting, the bottom 50 mm of the tube was not filled with concrete but with Styrofoam. The free space obtained after removing the Styrofoam made it possible to move concrete in relation to the FRP tube during the testing. The upper edge of the tube was equipped with a steel mold 40 mm in height, filled with a concrete mix. During the tests, the load was applied through this concrete part protruding from the FRP tube.

### 2.2. Structural Component Properties

**Figure 5:**
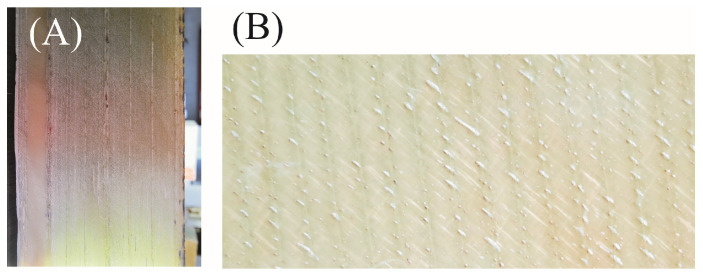
Specimen cut from spar cap: (**A**) cross-section, where internal layers are visible; (**B**) surface of FRP laminate.

In the cross-section of the FRP laminate of the spar cap, some layers can be distinguished (Figure 5A), in which the glass fibers are inclined to the longitudinal direction at angles of 45° and 135° (Figure 5B).

The material strength parameters were obtained from preliminary tests performed as part of this research.

The experiment to find the direct tensile strength of the FRP laminate was undertaken on specimens cut from box-shaped elements and prepared in accordance with standard ASTM D3039/D3039M [46]. Five specimens 7–11 mm in thickness cut from the sides of a spar cap were tested and failed in the middle, as recommended in the above standard. An average longitudinal tensile strength of 297 MPa, with a standard deviation of 52.9 MPa, was obtained.

The compressive strength of the FRP laminate was assessed with tests executed on six specimens of 50 × 50 mm dimensions and 23–24 mm thickness, cut from the longer walls of a spar cap. The average results differed depending on the direction of the fibers. When the load was applied parallel to the spar cap length, a compressive strength of 457.71 MPa with a standard deviation of 72.41 MPa and a modulus of elasticity of 39.57 GPa were obtained. When the load was perpendicular, a compressive strength of 121.36 MPa with a standard deviation of 21.68 MPa and a modulus of elasticity of 11.54 GPa were obtained.

The obtained tensile strength is comparable to that described in the literature results of pultruded GFRP tubes by Bazli et al. [44] (308.4 MPa), but lower than that obtained by Lin et al. [47] for GFRP pultruded plates of 12.7 mm thickness (537 MPa) and by Alshannaq et al. [14] for spar caps cut from turbine blades of a different type than that tested in the present study (597 MPa). The obtained compressive strength was between the results by Lin et al. [47] (377 MPa) and Alshannaq et al. [14] (504 MPa).

Concrete parameters were tested on the specimens prepared and cured in accordance with the EN 12390-1:2021-12 [48] and EN 12390-2:2019-07 [49] standards for 28 days and then tested. Three specimens of 150 × 150 × 150 mm dimensions were tested under compression (EN 12390-3:2019-07 [50]), three specimens of 150 × 150 × 150 mm dimensions were tested under tension (EN 12390-6:2011 [51]), and three cylindrical specimens of 150 × 300 mm were used to determine the secant modulus of elasticity in compression (EN 12390-13:2021-12 [52]). The average compressive strength was equal to 58.5 MPa with a standard deviation of 6.5 MPa, the modulus of elasticity was equal to 30.7 GPa, and the tensile strength equaled 3.8 MPa, with a standard deviation of 0.2 MPa.

### 2.3. Push-Out Test Execution

**Figure 6:**
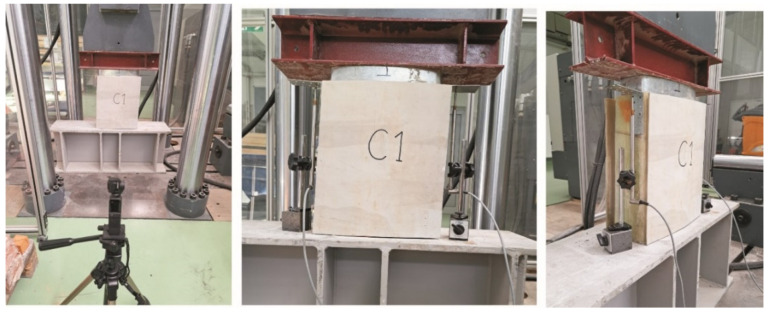
Execution of push-off test.

The push-out test was executed with the use of a Zwick-Roel testing machine (Figure 6). The load was applied to the specimens via a rigid steel structure. The value of the applied force was registered by the machine system, and the displacement of the concrete in relation to a composite tube was measured by two LVDT gauges placed on both sides of the specimen.

### 2.4. Pull-Off Test Execution

**Figure 7:**
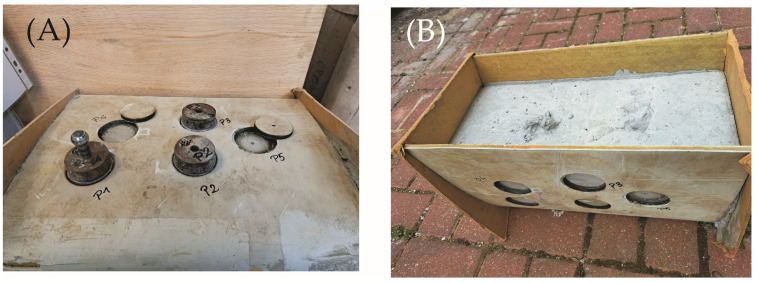
Pull-off test: (**A**) execution; (**B**) the specimen after testing.

The pull-off test was performed using Dynatest equipment (Figure 7). Before testing the adhesion of the laminate, the substrate was prepared by cleaning the surface and then cutting it with a crown with a diameter of 62 mm. Steel discs with a diameter of 50 mm were glued to the substrate with epoxy resin.

### 2.5. Numerical Analysis of Push-Out Test

**Figure 8:**
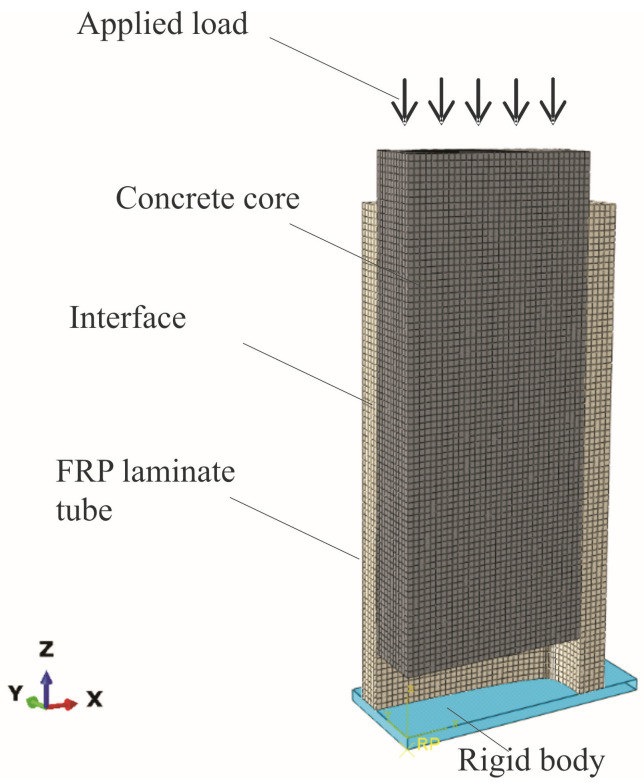
Discrete 3D model and applied boundary conditions of push-out test.

The push-out test model was built and analyzed using ABAQUS/Standard 6.12 software [53]. The finite element model of the specimen directly reflects the C4 specimen size. It was built using C3DR8 standard linear solid elements (Figure 8). The number of elements and nodes is listed in Table 2. The mesh size was approximately 5 mm, which was optimal for the model. Symmetry was assumed, and therefore a quarter of the specimen was modeled. Regarding the fact that the applied load was far lower than the compressive strength of both the FRP laminate tube and the concrete core (about 10%), for these materials, the elastic model was used in the range of the applied load (the elastic-ideal plastic for concrete and elastic-brittle for composite).

The following parameters of the materials (see Section 2.2 above) were assumed: composite longitudinal tensile strength—297.0 MPa, composite compressive strength—457.71 MPa, composite modulus of elasticity—39.57 GPa, concrete compressive strength—58.5 MPa, concrete tensile strength—3.8 MPa, and concrete modulus of elasticity—30.7 GPa.

**Figure 9:**
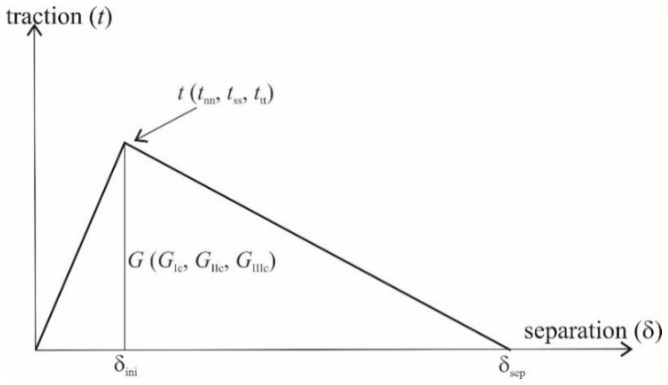
‘Traction–separation’ response assumed in FEM.

Similar to other models of multi-material structures (concrete and steel, two types of concrete), e.g., [54,55], adhesion between the laminate tube and the concrete core was adopted. Normal and tangential forces in the contact surface reflected their interaction, although the individual parts were independent. The ‘Hard’ contact type was assumed to describe the normal behavior, while friction was involved in the tangential behavior. The interface had negligibly small thickness, which is why the ‘surface-based cohesive method’ was used together with the ‘traction–separation’ law. The Abaqus law defines the initial linear elastic behavior followed by damage initiation and evolution (Figure 9). The elastic behavior is described in the form of an elastic constitutive matrix that relates the normal and shear stresses to the normal and shear slip at the interface. A damage model simulates the degradation following a failure of the bond between two cohesive surfaces. The failure mechanism relies on two agents: the damage initiation criterion, represented with the maximum slip value *δ*_ini_, and the damage evolution law, which corresponds to the complete separation of the connected surfaces when a slip of *δ*_sep_ is achieved. The nominal traction stress vector t consists of three components (in the case of two-dimensional problems—two components): *t*_n_, *t*_s_, and (in three-dimensional problems) *t*_t_. They represent the normal (along the local three directions in a three-dimensional model and along the local two directions in a two-dimensional model) and the two shear tractions (along the local one and two directions in a three-dimensional model and along the local one direction in a two-dimensional model), respectively. The accorded separations are denoted by *δ*_n_, *δ*_s_, and *δ*_t_. Therefore [53]:

(1) $$t=\left\{\left.\begin{array}{c} t_{nn}\\ t_{ss}\\ t_{tt} \end{array}\right\}\right.=\left[\begin{array}{cc} K_{nn} & \begin{array}{cc} K_{ns} & K_{nt} \end{array}\\ \begin{array}{c} K_{ns}\\ K_{nt} \end{array} & \begin{array}{cc} \begin{array}{c} K_{ss}\\ K_{st} \end{array} & \begin{array}{c} K_{st}\\ K_{tt} \end{array} \end{array} \end{array}\right]\left\{\left.\begin{array}{c} \delta_{n}\\ \delta_{s}\\ \delta_{t} \end{array}\right\}\right.=K\delta$$

where *t*, *t*_nn_, *t*_ss_, and *t*_tt_ are tractions in the cohesive surface, *K*, *K*_nn_, *K*_ss_, and *K*_tt_ are the cohesive surface stiffnesses, and *δ*, *δ*_n_, *δ*_s_, and *δ*_t_ are the separation displacements.

The following parameters of the interface were initially assumed:

- Cohesive surface displacement—*δ*_n_, *δ*_s_, and δ_t_ = 0.2 mm, in agreement with the recommendations given in [53];
- Traction stresses *t*_n_, *t*_s_, and *t*_t_ varied if the scope was 0.090–0.139 MPa, which corresponded to that obtained by Bazli et al. [44,45];
- Friction coefficient varied in the scope of 0.17–0.37, in agreement with Bazli et al.’s [44,45] findings;
- Stiffness of the cohesive surface *K* (*K*_nn_, *K*_ss_, and *K*_tt_) was set as the default.

The traction stress and coefficient of friction values were varied in individual probes of the parametric study in the ranges listed above in order to obtain the best fit for the experiment. The result of the study is presented in Section 3.3.

## 3. Results

### 3.1. Push-Out Test Results

**Figure 10:**
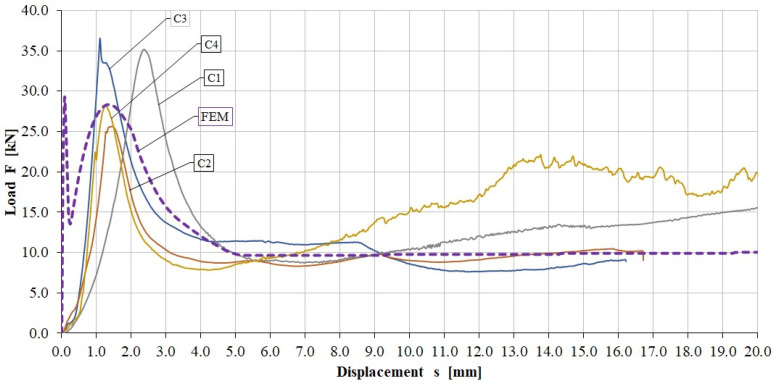
Experimental ‘load–displacement of concrete in relation to FRP tube’ relations; the dashed line represents the numerical analysis results (see Section 3.3, below).

The following findings were derived from the push-out test. The applied load increased regularly until the maximum was achieved. The peak value varied from 25.69 kN to 36.61 kN. The peak occurred while the displacement of concrete in relation to the FRP tube equaled 1.43–2.42 mm. Then, the load quickly diminished, and when the displacement equaled 4–5 mm, the load stabilized at about 8–20 kN. The characteristic values of the process are compiled in Table 3, whereas the ‘load–displacement of concrete in relation to FRP tube’ (‘load–slip’) relations are shown in Figure 10.

**Figure 11:**
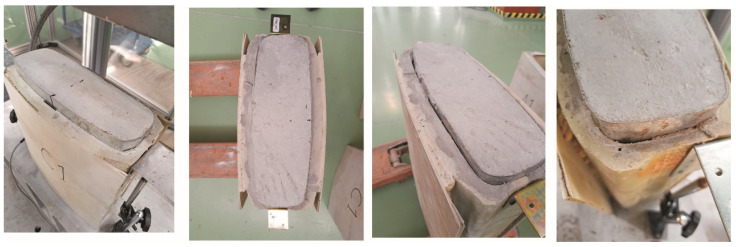
Post-testing push-out specimens.

The post-testing specimens are shown in Figure 11. The resulting discontinuities between the concrete and FRP laminate bear testimony to the adhesion breaking identified in Figure 10 with this load peak, followed by slippage of the concrete in relation to the FRP element.

### 3.2. Pull-Off Test Results

Five measurements were carried out, although only three reliable results were obtained—two of the composite disks broke off with the force value close to zero. The other results equaled 107 kPa, 256 kPa, and 170 kPa, resulting in an average value of 178 kPa. This can be regarded as the local tensile concrete bond strength of the tested FRP laminate, but with the significant caveat that the bond strength is not uniform across the interface, and locally, its value may be close to zero.

### 3.3. Results of Numerical Analysis

**Figure 12:**
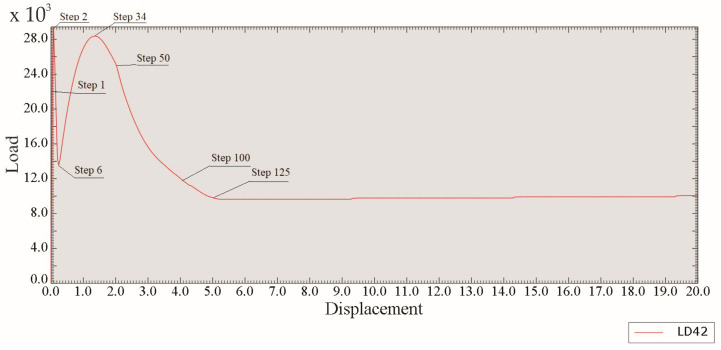
Response of the FEM specimen during the course of the push-off test.

The response of the numerical model, which reflects the highest extent of the course of the push-out test, is the ‘load–displacement of concrete in relation to FRP tube’ relation. This is the best information for the qualitative and quantitative analyses. While creating the relationship, the U3 axis displacement and R3 reaction force were taken into account. Figure 12 presents the ‘load–displacement’ curve obtained in the numerical analyses for a friction coefficient of *µ* = 0.22, a traction shear stress of *t*_n_ = *t*_s_ = *t*_t_ = 120 kPa, and a plastic displacement of *δ*_n_, *δ*_s_, *δ*_t_ = 0.2 mm. It was chosen from among the results of several probes of a parametric study executed on combinations of varying parameters as the best fit to the experimental results in the friction stage (see Figure 10).

The numerical curve has two peaks. The first is responsible for adhesion breaking and the second for overcoming the static friction. The same character was reported in [45]; however, the first peak was far lower than the second. In our tests, the specimen cross-section increased along the specimen height. This reduced the effective friction and caused the second peak to be almost on the same load level.

The fact that the first peak is not visible may be attributed to the fact that overcoming the static friction followed the adhesion breaking almost at once within the same time interval between measurements performed by the sensors of the testing machine. The lower joint stiffness demonstrated by experimental curves in comparison to numerical ones (almost vertical) is the result of the adaptation of the specimen to the testing machine conditions at the beginning of loading (to about 2–3 kN).

**Figure 13:**
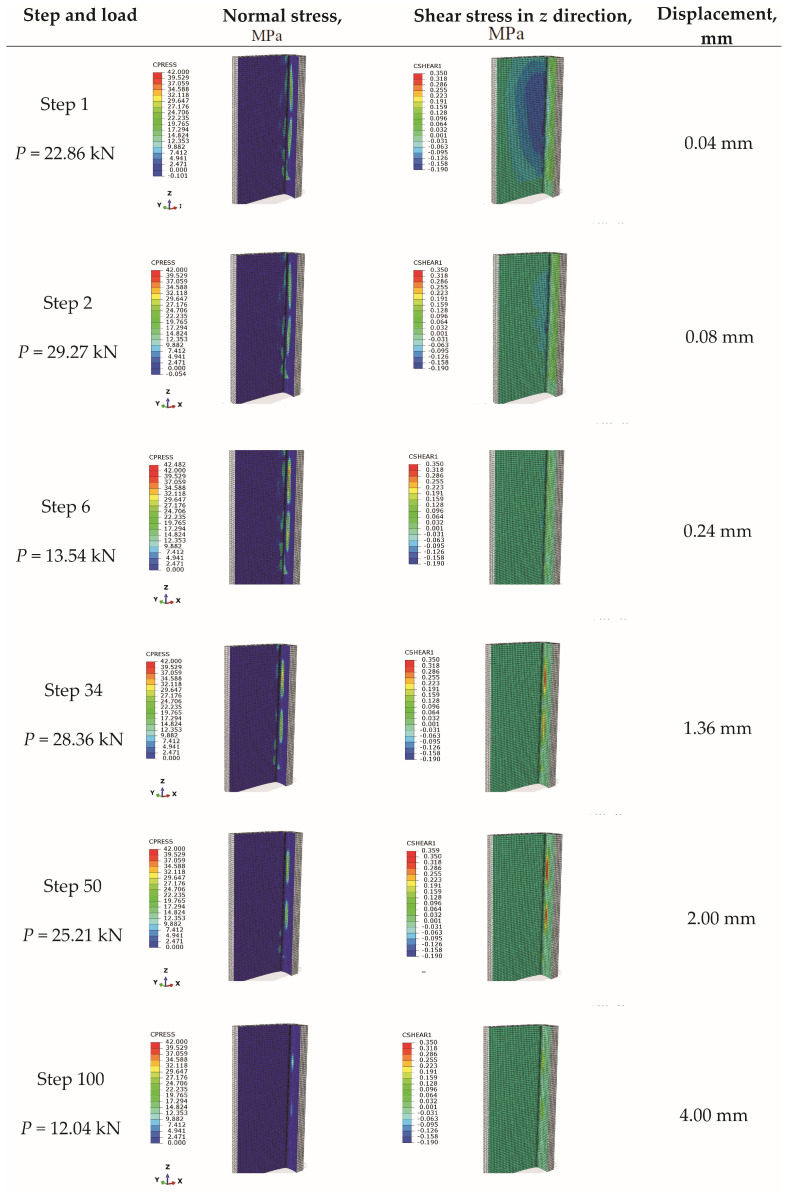
Stress distributions in the FEM specimen interface between the concrete and FRP tube, in MPa.

The stress distributions between the concrete and FRP tube, namely the shear stress in the vertical direction and normal stress, which were reported in the FEM interface, are presented in Figure 13. The six steps of the calculation, reflecting the interface performance, are shown as follows: Step (1) *P* = 22.86 kN, Step (2) *P* = 29.27 kN (before the adhesion breaking), Step (6) *P* = 13.54 kN, Step (34) *P* = 28.36 (maximum load), Step (50) *P* = 25.21 kN (maximum shear stress), and Step (100) *P* = 12.04 kN (when the process of breaking adhesion was almost finished).

In the numerical response, it can be observed that the damage to the interface (breaking the adhesion across the interface) is a progressive process. The short walls of the box-shaped FRP model tube are loaded in a higher extension than the longer ones. What is more, a concentration of stress is observed in the neighborhood of the specimen’s corner. In the ‘load–displacement’ curve (Figure 12), there is the characteristic peak of the load value related to a very small displacement of 0–0.08 mm (Step 1 and Step 2), which can be identified as the end of the ‘cohesive behavior’. At this stage of loading, a relatively uniform distribution of shear stresses over the entire surface of the laminate tube takes place. After breaking the adhesion (Step 2), elastic slipping occurs at the interface and the load decreases (Step 6). At this point, static friction comes into play and increases because the normal stress increases. The distributions of shear and normal stress are determined by the geometry of the concrete inner core and the form of its deformation under load. Therefore, the increase in normal and shear stresses is observed mainly on the short wall, which is deformed outwards in relation to the original core geometry.

It is noticeable that the maximum shear stress and maximum normal stress occur at different steps—the maximum normal stress is reached as early as at Step 6, while the shear stresses are reached at Step 50. This is due to the model assuming that elastic slip is allowed in the contact layer.

It is also characteristic that the shear stress reaches its maximum (Step 50) later than when the maximum load occurs (Step 34). After the static friction is overcome, the load gradually decreases to the stabilized level (Step 100), and the displacement increases without an increase in the load. Such behavior of the model can be attributed to the action of kinetic friction.

## 4. Discussion

First, it should be noted that the course of the obtained ‘load–displacement of concrete in relation to FRP tube’ relationships shown in Figure 7 is the same as obtained in other research concerning new (not recycled) GFRP tubes filled with concrete [41,42,43,44,45], as well as steel tubes filled with concrete [55].

In the analyses presented in [41,42,43,44,45], the concrete-to-laminate shear bond strength is regarded as the interface stress relating to the maximum force *P*_max_ that occurred during the push-off test. The abovementioned studies put it simply that the average shear bond strength constitutes the ratio of *P*_max_ and the interface surface area *A_i_*. This means that the assumption of a uniform distribution across the interface was adopted. In the present study, the first approximation of the shear bond strength value was similar, but the slope of the tube walls (expressed as the angle between the horizontal surface and walls—see Table 1) was regarded as:

(2) $$f_{b}=\frac{P_{max}sin\alpha }{A_{i}}.$$

The calculated average bond strength is shown in Table 4.

In Table 5, our results are compared with the results of other tests. The shear bond strength obtained in the present study (average 96 kPa) is higher than these obtained for new composite tubes by Ali [3] (22.5–30 kPa) but slightly lower than those obtained by Bazli et al. [17,18] (90–139 kPa). The slip related to the maximum shear stress is also comparable (1.35–1.46 mm in [17] and 1.54 mm in our tests).

However, the push-out tests of steel tubes filled with concrete [56] gave far higher bond strengths: 410–850 kPa in comparison to all FRP new and recycled tubes analyzed in Table 5. This proves that steel has a higher coefficient of friction than pultruded GFRP tubes. Tao et al. [57] tested steel tubes made of carbon and stainless steel (with differences in surface characteristics) of different cross-sections (circular and rectangular). They found a reduction in bond stress when stainless steel was used, which ranged from 32 to 69% in comparison to carbon steel (600–1840 kPa for carbon steel and 470–1010 kPa for stainless steel). The variations were the result of different slendernesses of tubes and different concrete strengths.

It should be noted that the tested specimens were not cylindrical. The cylindrical tubes were more effective than rectangular ones for the creation of the hybrid structure. The tendency may be derived from the tests of steel tubes [57]. The results of the stainless steel tubes were used in later deliberation regarding the shape influence because their smooth surface resembles the composite surface to a higher extent than the carbon steel surface. The bond strength varied from 470 to 1010 kPa for circular tubes and from 120 to 710 kPa for rectangular ones [57]. Therefore, the approximated average bond strength value obtained in our tests, equaling 97 kPa, does not diverge from either the results obtained for the GFRP cylindrical tubes (see Table 5) or from the rectangular tubes made of stainless steel, as described above.

The last parameter demanding the comment is the coefficient of friction. As shown in Section 3.3 for the result of the parametric study, the value of *μ* = 0.22 was obtained for the recycled FRP non-cylindrical tube. It was lower than that calculated on the basis of the measured surface strain of new GFRP tubes tested in [45]. The coefficient of friction was assessed as being equal to 0.26 for filament and 0.36 for pultruded cylindrical tubes.

It should be emphasized that due to the nonuniform distribution of stress along the specimens’ height and perimeter, the local shear bond stress may be higher than the average shear strength. Moreover, in the three-dimensional structures, the normal stress takes on an important role. Also, a simplification of the coefficient of friction calculated from Coulomb’s law using the average shear and normal stress may be burdened with error.

## 5. Conclusions

Based on the above tests and analysis, the following conclusions can be formulated:

1. The push-out testing shows that the average shear bond strength at the interface between the concrete and FRP tube obtained from the wind turbine blade spar caps was equal to 97 kPa. A numerical model of the tested tubes proved that the best fit to the experimental ‘load displacement of concrete in relation to FRP tube’ relationship was a shear strength (traction stress) equal to 120 kPa.
2. The coefficient of static friction between the concrete and FRP tube obtained from the wind turbine blade spar caps, assessed on the basis of the numerical results, gave a best fit of 0.22 for the experimental results.
3. The developed numerical model reflected the course of the push-out test in detail. The progressive character of interface destruction was reflected in the successive occurrence of adhesion breaking, building the static friction, overcoming the static friction, and the action of the kinetic friction at the end of the process.
4. The pull-off testing showed that although the tested average tensile bond strength between the concrete and FRP tube obtained from the wind turbine blade spar caps was quite high, at 178 kPa, regions existed where the tensile force was near zero. This proved that the adhesion is not uniform across the interface.
5. The presented research and previous authors’ tests [23] proved the cooperation in the load-bearing of the FRP element obtained from the dismantled turbine blade and concrete. Working out such structures should be based on numerical calculations regarding their 3D character and their exact shape. In the numerical parametric study, the interface parameters describing this cooperation were assessed as follows: a shear bond strength of 120 kPa and a coefficient of friction of 0.22.

The novelty of the presented research lies in proving that the reuse of large sections of wind turbine blades filled with concrete allows the creation of geotechnical structures. It is possible due to the cooperation between the reused composite and concrete, which was experimentally proved. The cooperation parameters (adhesion and coefficient of friction values) were found on the basis of tests and numerical analyses.

## Figures and Tables

**Table 1:** Internal dimensions of push-out specimens.

Specimen	C1	C2	C3	C4
Height *h*, [mm]	405	405	405	405
Upper length *l*_1,upper_, [mm]	310	333	321	311
Upper internal width *l*_2,upper_, [mm]	106	117	109	108
Upper internal perimeter *u*_upper_, [mm]	755	825	790	754
Upper internal area, [m^2^]	0.029	0.035	0.032	0.028
Bottom internal length *l*_1,bottom_, [mm]	320	344	329	323
Bottom internal width *l*_2,bottom_, [mm]	110	124	116	111
Bottom internal perimeter ubottom, [mm]	786	860	816	790
Bottom internal area, [m^2^]	0.032	0.039	0.035	0.033
Average slope of walls $\alpha =\mathrm{atan}\left[\frac{h}{0.25(u_{\mathrm{bottom}}-u_{\mathrm{upper}})}\right],[rad/deg]$	1.552/ 88.95	1.549/ 88.81	1.555/ 89.13	1.549/ 88.78
Internal area of walls *A*_i,_ [m^2^]	0.312	0.342	0.325	0.312

**Table 2:** Element type, the number of elements and nodes.

Component	FE Type	No. Elements	No. Nodes
Laminate	C3DR8 solid element	18,225	22,632
Concrete	C3DR8 solid element	28,598	32,480

**Table 3:** Characteristic values of the push-off process.

Specimen	C1	C2	C3	C4
Peak load, [kN]	35.14	25.69	36.61	28.11
Displacement related to peak value, [mm]	2.42	1.43	1.09	1.22
Lowest value of load, relating to slipping, [kN]	8.80	8.33	11.01	7.83

**Table 4:** Simplified calculation of average shear bond strength between concrete and laminar tube.

Specimen	C1	C2	C3	C4	Average
Peak load, [kN]	35.14	25.69	36.61	28.11	
Inner area of walls *A*_i_, [mm]	0.312	0.342	0.325	0.312	
Shear bond strength f_b_ in accordance with Equation (2), [kPa]	112.63	75.12	112.65	90.10	97.62
Lowest load value, characteristic of current friction stage, [kN]	8.80	8.33	11.01	7.83	
Shear interface stress *τ*_i_ related to friction after adhesion breaking, [kPa]	25.90	24.36	33.88	25.10	27.31

**Table 5:** Comparison of test results and concrete bond strength of pultruded GFRP tubes.

	Reference	Specimen Shape, Inner Dimension, [mm]	Length/ Thickness, [mm]	Average Bond Stress, [kPa]	Slip Related to Max. Stress, [mm]
push-out test	[42]	Tube ϕ279.6	200/12.7	30.0	0.84
	[42]	Tube ϕ380.6	200/12.7	22.5	0.42
	[44]	Tube ϕ78	200/8.0	90.0	1.35
	[44]	Tube ϕ166.2	330/8.6	130.0	1.46
	[45]	Tube ϕ86	200/8.0	139.0	
	Present study	Non-cylindrical tube 300 × 100	405/25	97.62	1.54

## Data Availability

The data presented in this study are available on request from the corresponding author due to (specify the reason for the restriction).
